# Systems and Synthetic microRNA Biology: From Biogenesis to Disease Pathogenesis

**DOI:** 10.3390/ijms21010132

**Published:** 2019-12-24

**Authors:** Hironori Matsuyama, Hiroshi I. Suzuki

**Affiliations:** 1 Fujii Memorial Research Institute, Otsuka Pharmaceutical Co., Ltd., 1-11-1 Karasaki, Otsu-shi, Shiga 520-0106, Japan; Matsuyama.Hironori@otsuka.jp; 2 David H. Koch Institute for Integrative Cancer Research, Massachusetts Institute of Technology, Cambridge, MA 02139, USA

**Keywords:** microRNA, RNAi, Drosha, Argonaute, biogenesis, super-enhancer, disease pathogenesis, synthetic biology

## Abstract

MicroRNAs (miRNAs) are approximately 22-nucleotide-long, small non-coding RNAs that post-transcriptionally regulate gene expression. The biogenesis of miRNAs involves multiple steps, including the transcription of primary miRNAs (pri-miRNAs), nuclear Drosha-mediated processing, cytoplasmic Dicer-mediated processing, and loading onto Argonaute (Ago) proteins. Further, miRNAs control diverse biological and pathological processes via the silencing of target mRNAs. This review summarizes recent findings regarding the quantitative aspects of miRNA homeostasis, including Drosha-mediated pri-miRNA processing, Ago-mediated asymmetric miRNA strand selection, and modifications of miRNA pathway components, as well as the roles of RNA modifications (epitranscriptomics), epigenetics, transcription factor circuits, and super-enhancers in miRNA regulation. These recent advances have facilitated a system-level understanding of miRNA networks, as well as the improvement of RNAi performance for both gene-specific targeting and genome-wide screening. The comprehensive understanding and modeling of miRNA biogenesis and function have been applied to the design of synthetic gene circuits. In addition, the relationships between miRNA genes and super-enhancers provide the molecular basis for the highly biased cell type-specific expression patterns of miRNAs and the evolution of miRNA–target connections, while highlighting the importance of alterations of super-enhancer-associated miRNAs in a variety of human diseases.

## 1. Introduction

MicroRNAs (miRNAs) are approximately 22-nucleotide-(nt)-long, small regulatory non-coding RNAs (ncRNAs) [1,2]. The first known miRNA was described in *C. elegans* in 1993 [3,4]. miRNA research was subsequently accelerated by the discovery of RNA interference (RNAi) in 1998 [5] and intensive molecular searches for similar endogenous small RNAs in various species in 2001 [6,7,8]. Thus far, more than 1800 and 1100 miRNA genes have been reported in humans and mice, respectively [9]. Further, miRNAs mediate post-transcriptional regulation of gene expression in a variety of species by recognizing multiple target mRNAs via sequence complementarity and (typically) repressing target RNAs. Numerous studies have demonstrated the widespread importance of miRNAs in development, normal physiology, and disease [1,10].

In this review, we summarize recent advances in miRNA research by focusing on the quantitative features of miRNA biogenesis and function; regulation by RNA modifications (epitranscriptomics), epigenetics, transcription factor circuits, and super-enhancers; and their applications for synthetic biology and the understanding of disease pathogenesis.

## 2. Overview of miRNA Biogenesis and Function

**Figure 1:**
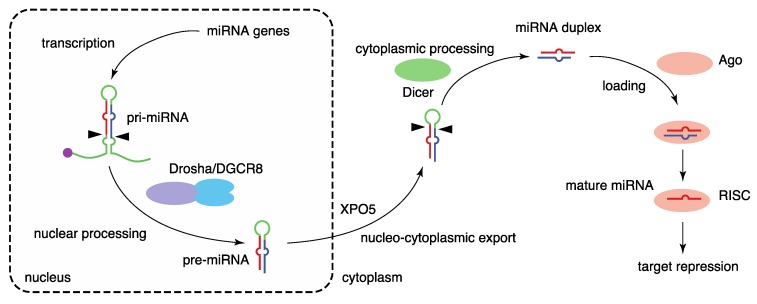
Biogenesis pathway of canonical miRNAs. miRNA biogenesis is mediated by multiple steps, including the transcription of primary miRNA transcripts, nuclear processing by Drosha, nucleocytoplasmic export by XPO5, cytoplasmic processing by Dicer, and formation of the RISC with Ago proteins.

The biogenesis of miRNA is mediated by multiple steps: transcription of primary miRNA transcripts, nuclear processing by Drosha, nucleocytoplasmic export, cytoplasmic processing by Dicer, and formation of RNA-induced silencing complex (RISC) with Argonaute (Ago) proteins [2,11,12,13] (Figure 1). The biogenesis of canonical miRNAs begins with the generation of long primary miRNA transcripts (pri-miRNAs), mainly by RNA polymerase II. Animal miRNAs are encoded as individual miRNA genes (monocistronic), as miRNA clusters (polycistronic), or in introns of protein-coding genes (intronic) [11,14]. Pri-miRNAs are processed to hairpin-structured RNAs, termed precursor miRNAs (pre-miRNAs), by the Drosha complex in the nucleus. The Drosha complex consists of the RNase III, Drosha, and the double-stranded RNA (dsRNA)-binding protein, DiGeorge syndrome critical region 8 (DGCR8), as well as various partner proteins. Following Drosha processing, the pre-miRNAs are exported to the cytoplasm by exportin-5 (XPO5). The pre-miRNAs are processed by the RNase III, Dicer, in the cytoplasm, liberating a 21–24 nt miRNA duplex. Several Dicer-associated proteins, including TRBP, PACT, and ADAR1, are known.

The miRNA duplex is next loaded into an Ago protein with assistance from the HSP70/HSP90 chaperone machinery to form RISC. Among the four mammalian Ago proteins (Ago1–4), only Ago2 has the potent slicer activity required for target mRNA cleavage in the small interfering RNA (siRNA) mechanism; the others have no slicer activity or have complicated substrate requirements to elicit slicer activity [15,16,17,18,19,20,21]. Of the two strands of miRNA duplexes, only one strand, termed the guide strand (referred to as miRNA), is retained in Ago proteins and stably forms the RISC. The other strand, known as the passenger strand (referred to as miRNA*), is discarded. The ratios of mature miRNAs derived the from 5′ (5p) and 3′ (3p) sequences of individual miRNA duplexes vary, and both strands are functional for some miRNAs [22]. To avoid confusion, mature miRNAs from the 5′ (5p) and 3′ (3p) arms are annotated with the suffixes -5p and -3p, respectively. The formation of the RISC stabilizes both miRNAs and Ago proteins. Several mechanisms of miRNA destabilization including Tudor-SN-mediated endonucleolytic decay and target-directed miRNA destabilization, which frequently involves 3′ end tailing and trimming and yields miRNA isoforms (isomiRs), have been described [23,24].

Finally, the Ago-miRNA complex binds predominantly to the 3′ untranslated region (3′ UTR) of target mRNAs in a sequence-specific manner and induces target repression with the aid of TNRC6 (GW182) proteins by shortening the poly(A) tails of mRNAs, repressing translation, and destabilizing mRNAs [12,25]. Target recognition of miRNAs typically depends on the seed sequence of miRNAs (nucleotides 2–7) [26,27]. Because the seed sequence is very short, one miRNA targets hundreds of target mRNAs, and it has been suggested that target repression is influenced by various RNA-binding proteins (RBPs) and other RNA molecules, such as circular RNAs, RNA sponges, and competing endogenous RNAs (ceRNAs) [28,29,30,31,32].

On the other hand, multiple Drosha- or Dicer-independent biogenesis routes have been characterized for several classes of non-canonical miRNAs, including mirtrons [33,34,35], tailed mirtrons [36], tRNA fragments [37,38], snoRNA fragments [39,40,41,42], 5′-capped miRNA precursor-derived 3p miRNAs [43], Dicer-independent erythrocyte-specific miR-451 [44,45], and transcription start site miRNAs (TSS-miRNAs) [46].

## 3. Quantitative Features of miRNA Biogenesis and Function

Recent studies have provided molecular and theoretical frameworks for a quantitative understanding of miRNA biogenesis and function, and a system-level understanding of miRNA networks. This section introduces recent topics in miRNA biogenesis and function.

Several studies using knock-out cells have reevaluated the relative importance of Drosha, Dicer, XPO5, TRBP, and PACT in miRNA biogenesis [47,48]. The production of most miRNAs, i.e., canonical miRNAs, is reportedly almost completely abolished in Drosha knock-out cells. Although Dicer knock-out cells also exhibited marked reductions of most miRNAs (>100-fold reduction of approximately 80% of miRNAs), many canonical miRNAs were still detectable [47]. In this setting, pre-miRNAs are loaded directly onto Ago proteins and trimmed at the 3′ end, producing 5p miRNAs. Thus, 3p miRNAs were more severely reduced than 5p miRNAs in Dicer knock-out cells. In contrast to Drosha and Dicer depletion, XPO5 depletion only modestly reduced the miRNA level, implying potential alternative transport mechanisms independent of XPO5. Another study showed that TRBP and PACT do not regulate miRNA abundance and strand selection [48], although TRBP depletion affected the accuracy of Dicer processing sites for several miRNAs and subsequently altered strand selection [48,49].

**Figure 2:**
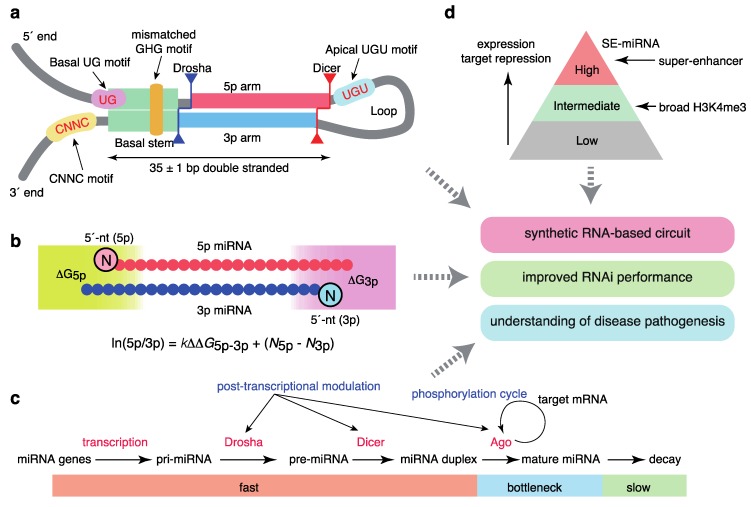
Quantitative features of miRNA homeostasis. (**a**) Sequence features that define pri-miRNAs. (**b**) Molecular principles of asymmetric strand selection. (**c**) Dynamics of miRNA metabolism and broad post-transcriptional regulation. (**d**) Relationships between super-enhancers and the hierarchy of the miRNA network.

Several studies have improved our understanding of how Drosha/DGCR8 process pri-miRNAs to pre-miRNAs (Figure 2a). Using high-throughput analyses of pri-miRNA variants, a number of sequence features of pri-miRNAs were shown to be important for efficient pri-miRNA processing; these features included a UG motif at the base of the pri-miRNA hairpin, a UGU(GUG) motif in the apical loop, a CNNC motif (SRp20/SRSF3-binding motif) 16–18 bp downstream of the Drosha processing site, a mismatched GHG motif in the basal stem region, and a narrow range of tolerable pri-miRNA stem lengths (35 ± 1 base pairs) [50,51]. These sequence features accommodate the structural features of the interaction between pri-miRNAs and the heterotrimeric Drosha/DGCR8 complex consisting of one Drosha and two DGCR8 [52,53]. Drosha interacts with the basal stem, recognizes a basal UG motif, and defines the processing site 11 and 13 bp from the basal ssRNA-dsRNA junction; DGCR8 interacts with the stem and apical regions and recognizes an apical UGU motif. In addition, the structural analysis of the Dicer complex with pre-miRNAs was recently reported [54].

The model for the mechanisms underlying the asymmetry of miRNA strand selection has been also revised recently (Figure 2b). By integrating systematic biochemical studies, structural insights into Ago proteins, and mathematical modeling, we demonstrated that small RNA asymmetry is directly driven by Ago proteins [55]. Ago2 selects strands with 5′-uridine or 5′-adenosine and thermodynamically unstable 5′ ends in parallel through its two sensor regions in the MID domain, which contact the 5′ nucleobases and 5′-phosphates of prospective guide strands. Thus, miRNA asymmetry shows superposed patterns reflecting 5′-end nucleotide identity and thermodynamic stability. Several reported features of small RNA asymmetry are consistent with our findings [56,57,58], and were integrated into a unified model. Based on enzyme kinetics modeling, the relative miRNA 5p/3p arm ratio can be described as follows:

ln(5p/3p) = *k*ΔΔ*G*_5p−3p_ + (*N*_5p_ − *N*_3p_) (1)

where Δ*G*_5p(3p)_ represents the thermodynamic stability of either end of the miRNA duplex, and *k* and *N*_5p(3p)_ represent the constant for relative thermodynamic stability and the constant corresponding to the 5′ end identity of the 5p (3p) strand, respectively. This model well explains the strand ratio assessed by next generation sequencing and the magnitudes of target repression by each strand obtained from reporter assays, as recently confirmed by another group [59].

Importantly, a recent time-resolved small RNA sequencing study using thiol (SH)-linked alkylation for the metabolic sequencing of RNA (SLAM-seq) has provided a quantitative view of miRNA production and destruction in *Drosophila* S2 cells (Figure 2c) [60]. The processing of pri-miRNAs and pre-miRNAs and generation of miRNA duplexes occurs in a matter of seconds or minutes, more rapidly than the generation of most mRNAs, and loading onto Ago proteins occurs approximately 1 h after the start of biogenesis, suggesting that Ago loading is a key bottleneck step for ensuring faithful miRNA production. miRNA degradation is typically slow; the median half-life is >10 h. These findings thus suggest that miRNAs are the most rapidly produced and longest-lasting cellular RNAs. A recent study in mammalian cells using 5-ethynyluridine labeling reported similar observations [61].

A number of protein modifications of biogenesis factors, including phosphorylation, are reported to modulate miRNA homeostasis [2,62]. A recent CRISPR/Cas9-based screening of miRNA modulators revealed that Ago2 is phosphorylated by CSNK1A1 and dephosphorylated by the ANKRD52–PPP6C phosphatase complex; moreover, phosphorylation of Ago2 inhibits target mRNA binding and target repression without a concomitant reduction in miRNA abundance (Figure 2c) [63]. Ago2 phosphorylation appears to occur at the late stages of target repression [63,64]. This phosphorylation cycle may be important for redirecting the RISC from one target mRNA to another, thereby maintaining the global efficiency of repression of hundreds of target mRNAs. Several reports have also described Ago2 phosphorylation by epidermal growth factor receptor (EGFR) and an interaction between KRAS and Ago2, suggesting widespread crosstalk between miRNA and cellular signaling pathways [65,66].

In addition, multiple reports have described the broad post-transcriptional mechanisms of miRNA processing mediated by various RBPs, including Lin28, and crosstalk between the pri-miRNA processing machinery and transcription-related machinery (Figure 2c) [2,13,62,67,68]. Two recent systematic surveys using a proteomics-based pull-down approach and enhanced UV crosslinking followed by immunoprecipitation (eCLIP) analysis have provided an expanded database of the RBPs that regulate miRNA biogenesis [69,70].

## 4. Integration of miRNA Biology and Synthetic Biology

A deeper understanding of miRNA biogenesis and function would contribute to the reduction of off-target effects and improvement of RNAi performance in both gene-specific targeting and genome-wide screening [71]. Furthermore, such improvements would facilitate the generation of synthetic RNA-based gene circuits in the field of synthetic biology [72]. Synthetic gene circuits are generated to process input information and produce a specific output. In this setting, miRNA pathways can be utilized for the programming of synthetic circuits. Further, miRNAs can be artificially incorporated into logic gates as internal components, while endogenous miRNAs can be utilized to sense specific cellular contexts as the input of circuits because of their highly biased cell type-specific expression patterns. The sequence features that define pri-miRNAs enable the optimization of efficient miRNA backbones, such as the miR-30 and miR-E backbones, and de novo design of functional miRNA genes (Figure 2a) [51,73]. The prediction of asymmetrical strand selection, presented in our previous study, is also able to enhance the utilization of the intended strands and reduce the off-target effects of passenger strands [55]. Nissim et al. developed a synthetic circuit by combining an RNA-based AND gate and de novo synthetic cancer-specific promoters that sense two transcription factors expressed in cancer cells, and adapted this circuit for cancer immunotherapy [74]. This RNA-based AND gate is based on the optimization of synthetic intronic pri-miRNAs, strand selection, and suppression of the auto-inhibitory loop by miRNA sponge RNAs. The circuit boosts the antitumor immune responses in vitro and in vivo, and therefore shows promise for increasing the efficacy and reducing the toxicity of engineered cell therapies, such as chimeric antigen receptor (CAR)-T cell therapy.

Endogenous miRNAs can also be used to classify cell types or identify cancer cells. As described later, miRNAs show highly biased cell type-specific expression patterns, and super-enhancers play central roles in cell type specificity. Several investigations have been performed attempting to develop a synthetic regulatory circuit that senses the expression levels of a customizable set of endogenous miRNAs [75,76,77]. This synthetic sensor triggers an artificial cellular response only when the expression pattern matches a predetermined profile, and has been recently adapted for cell type-specific CRISPR/Cas9-based genome regulation and cancer immunotherapy [78,79].

## 5. Roles of Epigenetics and Transcription in miRNA Expression

Changes in miRNA expression patterns have been characterized in various diseases, including cancer [80]. Further, miRNA expression is regulated during the transcription of pri-miRNAs, as well as during post-transcriptional processing and maturation [2,13,62]. While pervasive crosstalk between various RBPs and the miRNA processing machinery provides a broad layer of post-transcriptional regulation of pri-miRNAs and pre-miRNAs, the expression levels of mature miRNAs correlate with those of pri-miRNAs across diverse cell and tissue types, especially for highly differentially expressed miRNAs; this underscores the importance of cell type-specific transcription in organizing miRNA expression [81]. In the latter part of this review, we summarize recent advances in understanding the epigenetic and transcriptional regulatory mechanisms involved in miRNA expression by focusing on regulation mediated by (1) epigenetics, (2) transcription factors and transcription factor circuits, and (3) super-enhancers. We also introduce the roles of epigenetic modifications of RNAs (epitranscriptomics) in miRNA biology.

## 6. Regulation of miRNAs by RNA Modifications (Epitranscriptomics)

RNA modifications play important roles in RNA metabolism and modulate miRNA biogenesis and function; these modifications include alterations of N6-methyladenosine (m6A) and 7-methylguanosine (m7G), pseudourylation (ψ), and adenosine-to-inosine (A-to-I) editing. The conversion of adenosine to m6A in pri-miRNAs induced by the RNA methyltransferase, methyltransferase-like (METTL) 3, has been suggested to facilitate recognition and processing by DGCR8 [82,83]. Another report has described that m6A modifications by the tRNA methyltransferase, NSun2, suppress the miRNA processing of miR-125 [84]. In addition, m6A modifications are enriched at the 3′ UTRs and miRNA target sites of mRNAs, and miRNAs are reported to regulate m6A abundance by modulating METTL3 binding to mRNAs [85,86]. In contrast, phospho-dimethylation of the 5′ ends of pre-miRNAs by the RNA methyltransferase, BCDIN3D, reportedly inhibits Dicer processing [87].

A recent report described that METTL1 mediates m7G methylation of the G-quadruplex motif in pri-miRNAs of an important tumor suppressive miRNA, let-7e, leading to the suppression of G-quadruplex formation and enhancement of Drosha processing [88]; the authors of that study also reported a new method for detection of m7G methylation, which may provide insight into miRNA processing. A-to-I editing also affects the efficiency of Drosha and Dicer processing and recognition of miRNA targets by altering sequence complementarity within miRNA precursors and seed sequences [89,90,91]. Several recent studies have revealed distinct levels of A-to-I editing and m6A modifications of several miRNAs in various cancers, including lung adenocarcinoma and gastrointestinal cancer [92,93,94,95]. Using a non-targeted mass spectrometry sequencing technique for the unbiased detection of RNA modifications, one study demonstrated that the miR-17-5p m6A methylation level in serum samples could be used to distinguish patients with early-stage pancreatic cancer from healthy controls with higher sensitivity and specificity than CA19-9 and CEA, which are currently used in the clinic [95]. Although the biological importance of these disease-associated miRNA modifications is largely unclear, these modifications have potential for use as biomarkers to improve diagnosis and therapy.

## 7. Regulation of miRNAs by Epigenetics

Epigenetic regulation includes DNA methylation and chromatin/histone modifications, all of which modulate miRNA expression. The roles of epigenetic mechanisms in regulating miRNA expression have been reviewed by others [96,97]. In addition, miRNAs regulate various epigenetic regulators, thus establishing bidirectional crosstalk mechanisms [97].

A previous literature-based review suggested that approximately 120 miRNAs are epigenetically modulated in 23 cancer types, and that the methylation frequency of human miRNA genes appears to be much greater than that of protein-coding genes [98,99]. Consistent with this view, miRNA genes have been found to frequently overlap with CpG islands susceptible to methylation and with cancer-associated genomic regions [100,101]. The close proximity of pri-miRNAs to CpG islands is biased towards intergenic miRNAs, rather than intragenic miRNAs [100]. The methylation of CpG islands or residues typically reduces the activities of the host gene promoter, intronic miRNA promoter, miRNA gene promoter overlapping with or proximal to CpG islands, and distal enhancers, thereby resulting in differential miRNA expression.

In addition, histone modifications either activate or repress miRNA expression. An early study using breast cancer cell lines suggested that histone deacetylase (HDAC) inhibitors cause rapid and widespread changes in miRNA expression [102]. This rapid response suggests that epigenetic regulation affects co-transcriptional and/or post-transcriptional pri-miRNA processing, consistent with the existence of a super-enhancer-mediated pri-miRNA processing mechanism, as described later.

## 8. Regulation of miRNAs by Transcription Factor and miRNA Circuits

Transcription factors and miRNAs each alter the other’s expression, and it has been proposed that positive and negative transcriptional co-regulation circuits of a miRNA and its targets are prevalent in the mammalian system [103,104]. Several web tools and databases, including TFmiR, TransmiR, and CMTCN, have been developed to aid in the investigation of transcription factor-miRNA co-regulation [105,106,107]. In this section, we introduce the roles of signal transducer and activator of transcription 3 (STAT3) in miRNA regulation as a well-studied example in cancer biology, because STAT3 is the member of the STAT transcription factor family most frequently implicated in cancer biology. The STAT protein family is a cardinal component of the signaling cascades of various cytokines, including interferons and interleukin-6 (IL-6). Notably, STAT3 activation in cancer cells and cells of the tumor microenvironment has been linked to tumor promotion, suppression of anti-tumor immunity, and the inflammatory response in the tumor microenvironment [108,109]. STAT3 transcriptionally regulates multiple protein-coding genes and miRNA genes.

Persistent activation of STAT3 in cancer cells is attributable to autocrine or paracrine cytokine stimulation in the tumor microenvironment, expression of various oncogenic protein tyrosine kinases (e.g., Src) or oncogenic fusion proteins (e.g., nucleophosmin-anaplastic lymphoma kinase (NPM-ALK)), and mutation of STAT3 pathways [110,111,112,113]. We and others have described the effects of the NPM-ALK/STAT3 axis on miRNA expression in ALK-positive anaplastic large cell lymphoma (ALCL). Our findings demonstrated that NPM-ALK/STAT3-driven miR-135b potentiates tumor progression via multiple targets—including FOXO1, STAT6, GATA3, and PPP2R5C—in ALK-positive ALCL [114,115]. As a unique mechanism, miR-135b suppresses two master regulators of T-helper (Th) 2 differentiation, STAT6 and GATA3, and miR-135b blockade suppresses IL-17 production and paracrine inflammatory response by ALCL cells [114]. These results suggest that miR-135b-mediated Th2 suppression exerts broad effects on the ALCL immunophenotype, including bias toward a Th17-like phenotype. This type of non-cell-autonomous role of cancer-related miRNAs in the tumor microenvironment has been further reinforced by multiple other studies [116]. Other reports have reported that the NPM-ALK/STAT3 pathway induces downregulation of miR-26a targeting inducible nitric oxide synthase (iNOS), miR-29a targeting MCL1, miR-150 targeting MYB, and miR-219 targeting CD278 (also known as ICOS), and upregulation of oncogenic miR-17/92 cluster targeting BIM and transforming growth factor-β (TGF-β) type II receptors (TβRII) [117,118,119,120,121,122]. NPM-ALK is also presumed to modulate several miRNAs via other transcription factors, including C/EBPβ [123].

Furthermore, miRNAs are known to play important roles downstream of the IL-6/STAT3 signaling axis in various cancer types [124,125]. It has been repeatedly reported that STAT3 directly activates oncogenic miR-21 (Table 1). In multiple myeloma, this activation involves a highly conserved enhancer upstream of the miR-21 gene promoter [124]. The activation of several miRNAs (e.g., miR-21 and miR-181-b1) by the IL-6/STAT3 axis has been proposed to maintain the transformed state by increasing NF-κB activity through suppression of PTEN and CYLD tumor suppressors in diverse cell lines, thus forming a positive feedback loop linking inflammation to cancer [126]. A similar inflammatory feedback regulation involving STAT3, HNF4α, IL6R, miR-124, miR-24, and miR-629 was reported in hepatocellular carcinoma [127]. In addition, the STAT3-mediated repression of miR-34a and targeting of IL6R by miR-34a comprises a feedback loop required for IL-6-induced epithelial–mesenchymal transition (EMT) in colorectal cancer [128]. In addition, STAT3-suppressed miR-218 targets various upstream and downstream components of receptor tyrosine kinase (RTK) signaling, thereby promoting RTK signaling in glioblastoma [129]. In contrast, STAT3-mediated induction of miR-146b suppresses NF-κB-dependent IL-6 production and forms a negative feedback loop to limit STAT3-driven oncogenic phenotypes [130]. However, this negative feedback circuit is blunted by increased methylation of the miR-146b promoter in breast cancer [130]. The literature regarding STAT3-regulated miRNAs is summarized in Table 1 [131,132,133,134,135,136,137,138,139,140,141,142,143,144,145,146,147,148,149,150,151,152,153,154,155,156,157,158,159,160,161,162,163,164,165].

## 9. Regulation of miRNAs by Super-Enhancers

A few transcription factors, known as master transcription factors (e.g., Oct4, Sox2, Nanog, and Klf4 in embryonic stem cells (ESCs)), are essential for the establishment and maintenance of the identity of each cell type. Cell type-specific transcriptional programs are mediated by the activities of cell type-specific enhancers bound by transcription factors, and master transcription factors bind to thousands of enhancer regions, which can be identified by chromatin immunoprecipitation-sequencing (ChIP-seq) technologies. However, a few hundred large enhancer regions near cell identity genes consist of clusters of enhancer elements occupied by exceptionally high densities of master transcription factors [166,167]. Such enhancer domains, known as super-enhancers (SEs), are densely occupied by Mediator complexes and bear high densities of active chromatin markers, such as H3K27ac. In contrast to typical enhancers, super-enhancers show high transcriptional activity and marked vulnerability to the depletion of master transcription factors and transcription coactivators, including Mediator and Brd4.

These features of SEs and typical enhancers correspond to those of cell type-specific miRNA expression (Figure 2d). Although about 100 miRNAs show some evidence of expression in one cell type, a few abundant miRNAs dominate miRNA-guided post-transcriptional regulation from the standpoint of expression, Ago2 binding, and target repression [32,104,168]. Via the integrated analysis of the relationships between miRNAs and SEs, we reported that SEs are linked to a few highly abundant and tissue-specific miRNAs and master transcription factors [169]. The SE-associated miRNAs (SE-miRNAs) include most miRNAs for which depletion results in developmental abnormalities in the respective tissues. Further, their targets are associated with cell type-specific functions and transcriptional regulation, suggesting an intimate interplay between transcription factors and SE-miRNAs [169]. To ensure tissue-specific gene expression programs, it has been suggested that genes coexpressed with specific miRNAs avoid miRNA sites (target avoidance phenomenon), and that miRNAs and their targets show mutually exclusive expression patterns [170,171]. We revealed that the depletion of miRNA sites in coexpressed genes is positively correlated with the connection between SEs and miRNA genes [169]. This observation is consistent with the strictly conserved relationships among STAT3, miR-21, and STAT3-bound miR-21 enhancers [124], as described above, collectively suggesting co-evolution of the network involving transcription factors, enhancers, and miRNAs in development. While SEs can be identified by the reanalysis of ChIP-seq data and several regulatory regions have been proposed (e.g., stretch enhancers), our analysis supports SEs as the major drivers of cell identity. Indeed, a recent report demonstrated that SEs are more transcriptionally active and cell type-specific than stretch enhancers [172].

In addition, we have reported several unique mechanistic aspects of SEs [169]: (1) multiple SE constituents drive cell type-specific miRNAs in a cooperative manner, consistent with other functional studies of SEs [173,174]; (2) SEs are associated with chromatin recruitment of DGCR8 and Drosha, and facilitate pri-miRNA processing; (3) Drosha-enhanced mRNA degradation events are associated with DGCR8-dependent suppression of chromatin-associated SE-associated gene products; and (4) the bromodomain and extraterminal (BET) domain inhibitor, JQ1, inhibits chromatin DGCR8/Drosha recruitment at SEs and pri-miRNA processing of SE-miRNAs. These features are consistent with a recent phase separation model of SEs [175].

## 10. Super-Enhancer-Associated miRNAs in Disease

Disease-associated genome variation identified by genome-wide association studies (GWAS) is frequently found in SEs of disease-relevant cell types [167]. In addition, multiple mechanisms contribute to the SE activation responsible for the activation of multiple oncogenes in cancer. We have described the relationship between SE-miRNAs and cancers [169]. Loss and gain of SEs have been frequently found in the neighborhoods of tumor-suppressive and oncogenic miRNAs, respectively; miRNAs with SE alterations were linked to wide aspects of cancer hallmarks [176]. In addition, miRNAs with SE gain were associated with a worse prognosis. A recent report described the relationship between the Chr19q13.41 miRNA cluster (C19MC), encoding 54 miRNAs normally expressed in placental and germinal tissues, and SEs in highly lethal type of infant brain cancer, embryonal tumors with multilayered rosettes (ETMRs) [177]. ETMRs are characterized by the amplification of C19MC. High expression levels of C19MC, LIN28A, and MYCN comprise an oncogenic circuit in this tumor type [177]. This oncogenic circuit is reinforced by an enhancer hijacking mechanism: the formation of hybrid SEs via C19MC–TTYH1 gene fusion, which juxtaposes TTYH1-associated SEs and C19MC-associated enhancers located in distinct loci of chromosome 19. Additional long-range DNA interactions involving MYCN and neighboring SEs also contribute to the C19MC–LIN28A–MYCN oncogenic circuits. Reflecting the high dependency on SEs, JQ1 suppresses the expression of SE-associated oncogenes and C19MC miRNAs in the EMTR cells.

Our recent report has further demonstrated unique involvement of mutations of SE-miRNAs in human rare disease [178]. Grigelioniene et al. identified a neomorphic seed region mutation in the chondrocyte-specific SE-associated miR-140 gene (chr16:g.69967007A>G (hg19), MIR140:NR_029681.1:n.24A>G) in a novel skeletal dysplasia (spondyloepiphyseal dysplasia (SED) *MIR140* type Nishimura) [178]. While miR-140-null mice showed short stature and craniofacial abnormalities, mice with the corresponding mutation exhibited additional skeletal abnormalities similar to those observed in human patients. Transcriptome analysis unveiled both widespread derepression of wild-type miR-140-5p targets and repression of the targets of mutant miR-140-5p (miR-140-5p-G) in chondrocytes, suggesting both loss-of-function and gain-of-function effects. While heterozygous loss-of-function point mutations of miRNA genes (miR-96 and miR-184) have been reported in several congenital diseases (e.g., autosomal dominant deafness 50 and endothelial dystrophy, iris hypoplasia, congenital cataract, and stromal thinning (EDICT) syndrome) [179,180,181,182,183], the report involving miR-140 is the first report of a gain-of-function mutation of a miRNA gene in human disease. Because the magnitude of miRNA-mediated target repression is typically small, and the biological roles are thought to be stabilized by co-evolution of miRNA-target relationships, one may assume that a neomorphic mutant miRNA would need to target a biologically important regulatory network in order to produce a disease phenotype. To explain this, we found that the target sequence of the miR-140-5p-G seed overlaps with the binding motif of the conserved RBP Ybx1, and that miR-140-5p-G competes with Ybx1 for overlapping binding sites. We investigated whether this type of seed–RBP crosstalk, termed cross-talk with endogenous RNA-binding protein (ceRBP), contributes to off-target activities of RNAi; we found that ceRBP effects are observed for many RBPs and affect RNAi performance [184]. Given that RNA modifications, such as m6A and A-to-I editing, are targeted toward specific sequence motifs, modifications of miRNAs may converge on crosstalk with specific RBPs.

## 11. Conclusions and Perspectives

In this review, we summarized recent advances in the miRNA field regarding: (1) the quantitative understanding of miRNA biogenesis with respect to sequence and structural features, (2) the roles of epitranscriptomics (RNA modifications), epigenetics, and transcription factor circuits in miRNA regulation, (3) the roles of super-enhancers in miRNA regulation, and (4) the applications of these findings for synthetic biology, optimization of RNAi, and an understanding of disease pathogenesis (Figure 2). These advances collectively facilitate a system-level understanding of the broad miRNA network that programs cell-type specificity and mediates the pathogenesis of diverse diseases. The quantitative programming of miRNA function and identification of SE-miRNAs would facilitate the manipulation of specific cell populations and engineering of artificial cellular functions in regenerative medicine and synthetic biology.

With respect to clinical applications, the miRNA-based drugs Miravirsen (a miR-122 inhibitor) and MRX34 (a miR-34a mimic) have been subjected to clinical trials [185,186,187]. In addition, the siRNA-based agent Onpattro™ was approved for the treatment of transthyretin familial amyloid polyneuropathy (ATTR-FAP) in 2018 [188], as was the anti-sense drug Tegsedi™ [189]. This was the first approved siRNA-based drug. Continuous efforts towards understanding the biology, improving molecular tools, and developing new technologies will provide a basis for the development of miRNA/RNAi-based diagnostic and therapeutic approaches [190].

## Figures and Tables

**Table 1:** Summary of miRNAs regulated by STAT3.

STAT3-Regulated miRNA	Disease or Target Cell	Change in Expression	miRNA Target	(Potential) Function and Phenotype	References
let-7b, -7c, -7e, -7g	Breast cancer	Downregulation ^1^	HMGA2	EMT	[131]
miR-200b/c	Breast cancer	Downregulation	ZEB1	EMT	[131]
miR-106a	Ovarian cancer	Upregulation	PTEN	proliferation and invasion	[132]
miR-125b-2, -30c-1, -23b/27b/24-1, -17/92	Toxoplasma gondii infection	Upregulation	-	anti-apoptosis	[133]
miR-135b	ALK-positive ALCL	Upregulation	GATA3, STAT6, FOXO1, PPP2R5C	modulation of tumor immune-phenotype, tumor microenvironment, and chemotherapeutic resistance	[114,115]
miR-143	Blood brain barrier damage induced by methamphetamine	Upregulation	PUMA	modulation of tight junction proteins	[134]
miR-146a	Hepatocellular carcinoma	Upregulation	STAT1, TRAF6	immunosuppressive tumor microenvironment	[135]
miR-146b	Breast cancer	Downregulation ^2^	IRAK1, TRAF6	NF-kB/IL-6/STAT3 negative feedback loop	[130]
miR-150	ALK-positive ALCL	Downregulation ^2^	MYB	proliferation	[119]
miR-155	Th17 cell	Upregulation	-	development of experimental autoimmune uveitis	[136]
miR-155	Chronic lymphocytic leukemia (CLL)	Upregulation	-	-	[137]
miR-155	Acute myelogenous leukemia (AML)	Downregulation	SOCS1	cell viability and myeloid differentiation	[138]
miR-155, -21, -15a, -16, -181a	CLL	Upregulation	-	-	[139]
miR-17/92 cluster	Pulmonary arterial hypertension	Upregulation	BMPR2	vascular remodeling	[140]
miR-17/92 cluster	ALK-positive ALCL	Upregulation	BIM, TβRII	anti-apoptosis	[121]
miR-181a	Triple-negative breast cancer (TNBC)	Upregulation	BAX	anti-apoptosis, chemotherapeutic resistance, and metastasis	[141]
miR-181b	Eophageal cancer stem-like cell	Upregulation	CYLD	proliferation and anti-apoptosis	[142]
miR-21	Transformed cell (Colon cancer)	Upregulation	PTEN	maintenance of transformed state	[126]
miR-181b-1	Transformed cell (Colon cancer)	Upregulation	CYLD	maintenance of transformed state	[126]
miR-183/96/182 cluster	Breast cancer	Upregulation	BRMS1L, GHR	EMT and invasion	[143]
miR-182-5p	Glioma	Upregulation	PCDH8	proliferation and invasion	[144]
miR-184	Keratinocyte	Upregulation	AGO2	cytokine-dependent Ago2 suppression	[145]
miR-197	Keratinocyte	Upregulation	IL22RA1	negative feedback loop of IL-22 signaling	[146]
miR-200c	Breast cancer	Downregulation ^2^	OBR	cancer stem cell plasticity	[147]
miR-204	Pancreatic beta cells	Downregulation	MAFA	insulin production	[148]
miR-204-5p	Endometrial carcinoma	Downregulation	TrkB	growth, migration, and invasion	[149]
miR-204	EBV-associated nasopharyngeal carcinoma	Downregulation	Cdc42	invasion and metastasis	[150]
miR-21	Multiple myeloma	Upregulation	-	-	[124]
miR-21	Heart failure	Upregulation	-	-	[151]
miR-21	ALK-positive ALCL	Downregulation	DNMT1	suppression of IL2Rγ	[152]
miR-21	Coronary artery endothelial cell	Upregulation	-	angiogenesis	[153]
miR-21	Hepatocellular carcinoma	Upregulation	-	HBV-induced transformation	[154]
miR-21	Alcoholic liver disease	Upregulation	FASLG (CD95L), DR5	survival, transformation, and liver fibrosis	[155]
miR-21	Nasopharyngeal carcinoma	Upregulation	PTEN	proliferation and anti-apoptosis	[156]
miR-214	Ulcerative Colitis	Upregulation	PTEN, PDLIM2	inflammation, colitis, and progression to colorectal cancer	[157]
miR-218	Glioblastoma	Downregulation	RSK2, S6K1, PDGFRα	regulation of RTK signaling	[129]
miR-219	ALK positive ALCL	Downregulation	CD278 (ICOS)	proliferation	[120]
miR-22	Cutaneous T cell lymphoma (CTCL)	Downregulation	NCOA1, PTEN, MAX	tumor progression	[158]
miR-23a	Hepatocellular carcinoma	Upregulation	G6PC, PGC1α	suppression of gluconeogenesis	[159]
miR-24, miR-629	Hepatocellular carcinoma	Upregulation	HNF4α	inflammation and tumor progression	[127]
miR-26a	ALK-positive ALCL	Downregulation	iNOS	cell viability, adhesion, and migration	[117]
miR-29a	ALK-positive ALCL	Downregulation ^2^	MCL1	anti-apoptosis	[118]
miR-29a, -29b, -29c	CD4 T cells under HIV-1 infection	Upregulation	HIV-1 mRNA	CD4 T cell-intrinsic resistance to HIV-1 infection	[160]
miR-34a	Colorectal cancer	Downregulation	IL6R	EMT and invasion	[128]
miR-34a	Breast cancer	Downregulation	Wnt1	tumor progression	[161]
miR-370	Wilms tumor	Upregulation	WTX	proliferation	[162]
miR-383	Skin cancer	Downregulation	ATR	anti-apoptosis	[163]
miR-520d-5p	Gastric cancer	Downregulation	CypB	proliferation	[164]
miR-92a	Lung cancer	Upregulation	RECK	invasion	[165]

^1^ Downregulation mediated by Lin28. ^2^ Downregulation mediated by DNA methylation.

## Abbreviations

3′ UTR	3′ untranslated region
A-to-I	adenosine-to-inosine
Ago	Argonaute
ALCL	anaplastic large cell lymphoma
AML	acute myelogenous leukemia
ATTR-FAP	transthyretin familial amyloid polyneuropathy
BET	bromodomain and extraterminal
C19MC	Chr19q13.41 miRNA cluster
CAR	chimeric antigen receptor
ceRBP	cross-talk with endogenous RNA binding protein
ceRNA	competing endogenous RNA
ChIP-seq	chromatin immunoprecipitation-sequencing
CLL	chronic lymphocytic leukemia
CTCL	cutaneous T cell lymphoma
DGCR8	DiGeorge syndrome critical region 8
dsRNA	double-stranded RNA
eCLIP	enhanced UV crosslinking followed by immunoprecipitation
EDICT	endothelial dystrophy, iris hypoplasia, congenital cataract, and stromal thinning
EGFR	epidermal growth factor receptor
EMT	epithelial–mesenchymal transition
EMTR	embryonal tumors with multilayered rosettes
ESC	embryonic stem cell
GWAS	genome-wide association study
HDAC	histone deacetylase
IL-6	interleukin-6
iNOS	inducible nitric oxide synthase
m6A	N6-methyladenosine
m7G	7-methylguanosine
METTL	methyltransferase-like
miRNA	microRNA
ncNRA	non-coding RNA
NPM-ALK	nucleophosmin-anaplastic lymphoma kinase
pre-miRNA	precursor miRNA
pri-miRNA	primary miRNA
RBP	RNA-binding protein
RISC	RNA-induced silencing complex
RNAi	RNA interference
RTK	receptor tyrosine kinase
SE	super-enhancer
SE-miRNA	super-enhancer-associated miRNA
SED	spondyloepiphyseal dysplasia
siRNA	small interfering RNA
SLAM-seq	Thiol (SH)-linked alkylation for the metabolic sequencing of RNA
STAT3	signal transducer and activator of transcription 3
TGF-β	transforming growth factor-β
Th	T-helper
TNBC	triple-negative breast cancer
TSSmiRNA	transcription start site miRNA
TβRII	TGF-β type II receptor
XPO5	exportin-5
